# Impact of App-Delivered Mindfulness Meditation on Functional Connectivity, Mental Health, and Sleep Disturbances Among Physician Assistant Students: Randomized, Wait-list Controlled Pilot Study

**DOI:** 10.2196/24208

**Published:** 2021-10-19

**Authors:** Jeremy L Smith, Jason W Allen, Carla I Haack, Kathryn L Wehrmeyer, Kayley G Alden, Maha B Lund, Jennifer S Mascaro

**Affiliations:** 1 Department of Radiology and Imaging Sciences Emory University Atlanta, GA United States; 2 Department of Neurology Emory University Atlanta, GA United States; 3 Department of Surgery Emory University Atlanta, GA United States; 4 Department of Family and Preventative Medicine Emory University Atlanta, GA United States; 5 Physician Assistant Program Department of Family and Preventative Medicine Emory University Atlanta, GA United States

**Keywords:** mindfulness, meditation, resting state, fMRI, connectivity, mobile phone

## Abstract

**Background:**

Health care provider and trainee burnout results in substantial national and institutional costs and profound social effects. Identifying effective solutions and interventions to cultivate resilience among health care trainees is critical. Although less is known about the mental health needs of physician assistants (PAs) or PA students, accumulating research indicates that they experience similarly alarming rates of burnout, depression, and emotional exhaustion. Mobile app–delivered mindfulness meditation may be an effective part of salubrious programming to bolster long-term resilience and health among PA students.

**Objective:**

This study aims to examine the impact of app-delivered mindfulness meditation on self-reported mental health symptoms among PA students. A secondary aim is to investigate changes in brain connectivity to identify neurobiological changes related to changes in mental health symptoms.

**Methods:**

We recruited PA students enrolled in their third semester of PA school and used a longitudinal, randomized, wait-list–controlled design. Participants randomized to the mindfulness group were provided 1-year subscriptions to the *10% Happier* app, a consumer-based meditation app, and asked to practice every day for 8 weeks. Before randomization and again after completion of the 8-week program, all participants completed resting-state functional magnetic resonance imaging as well as self-report assessments of burnout, depression, anxiety, and sleep impairment. App use was acquired as a measure of mindfulness practice time.

**Results:**

PA students randomized to the mindfulness group reported improvements in sleep impairment compared with those randomized to the wait-list control group (η_p_^2^=0.42; *P*=.01). Sleep impairment decreased significantly in the mindfulness group (19% reduction; *P*=.006) but not in the control group (1% reduction; *P*=.71). There were no other significant changes in mental health for those randomized to app-delivered mindfulness. Across all students, changes in sleep impairment were associated with increased resting-state functional connectivity between the medial prefrontal cortex (a component of the default mode network) and the superior temporal gyrus, as well as between areas important for working memory. Changes in connectivity predicted categorical conversion from impaired to nonimpaired sleep in the mindfulness group.

**Conclusions:**

This pilot study is the first to examine app-based mindfulness for PA students’ mental health and investigate the impact of mindfulness on PA students’ brain function. These findings suggest that app-delivered mindfulness may be an effective tool to improve sleep dysfunction and that it may be an important part of the programming necessary to reduce the epidemic of suffering among health profession trainees.

## Introduction

### Background

Although the high prevalence of depression among medical students and residents is well characterized and increasingly appreciated [1,2], little is known about the mental health of physician assistant (PA) students [3,4]. The research that has been conducted indicates that practicing PAs report high levels of burnout [5] and depression [6], and this is despite the fact that PAs often report high levels of job satisfaction [6]. Although very few studies have evaluated burnout or depression among PA students, one recent study found that almost 80% of PA students report high levels of emotional exhaustion, with almost that many expressing interest in interventions to improve their well-being [7]. Overall, there is a critical need to examine the mental health needs of PA students and evaluate interventions to bolster PA student resilience.

A substantial and growing body of research indicates that mindfulness meditation enhances well-being [8]; reduces anxiety and depression [9]; and optimizes immune signaling, stress responsivity, and cognitive function [10-12]. Moreover, mindfulness meditation has shown great promise for improving sleep disruption and insomnia symptoms [13-15], which may be beneficial to health professional trainees who often experience high rates of sleep dysfunction [16]. With its apparent efficacy, several clinical trials have examined whether mindfulness training improves well-being among health care trainees, including medical and nursing students and medical residents [17]. Accumulating research indicates that mindfulness reduces anxiety and depression and enhances well-being among health profession trainees, and a recent meta-analysis highlighted the potential efficacy of mind-body wellness programs such as mindfulness meditation for improving trainee well-being [18,19]. However, most studies examining mindfulness among trainees have examined time-intensive interventions that are prohibitive for most trainees. Although a recent study found that incorporating mindfulness into PA coursework increased self-reported well-being [20], very few studies have examined the impact of mindfulness training on the mental health of PA students.

Even fewer studies have examined the impact of mindfulness on trainee brain function. Growing evidence indicates that the health-relevant effects of mindfulness are mediated by alterations to the default mode network (DMN), the salience network (SN), and the systems involved in executive control, often referred to as the central executive network [21,22]. In addition, at least 3 studies indicate that mindfulness training [23,24] or dispositional mindfulness [25] are related to increased functional connectivity between the prefrontal cortex and the amygdala, generally interpreted as augmented emotion regulation via top-down control of the amygdala. Although these studies indicate that benefits from mindfulness meditation are related to changes in functional connectivity within and among these brain regions, to date, no studies have examined changes in functional connectivity related to improvements in well-being among health profession trainees.

### Objective

Here, we use a longitudinal, randomized wait-list–controlled design to examine the impact of app-delivered mindfulness meditation on self-reported mental health symptoms among PA students. A second aim is to examine whether changes in mental health are associated with changes in brain connectivity, indexed using a whole-brain connectome and multivariate pattern analysis approach to query within- and between-network connectivity across the entire brain before and after mindfulness training. On the basis of prior studies, we hypothesize that app-delivered mindfulness would reduce burnout, depression, anxiety, and sleep impairment; that changes in mental health outcomes would be associated with changes in DMN, SN, and central executive network connectivity; and that changes in mental health and connectivity would be positively associated with practice time, indexed as app use.

## Methods

### Recruitment

Students enrolled in their third semester of PA school were recruited for the study just before the commencement of their clinical rotations. Students were recruited via in-person presentations held after their classes, and 16 students were enrolled in the study. Upon providing informed consent in accordance with the university’s institutional review board, participants were randomly assigned (in Microsoft Excel, using the *randbetween* function) to either a mindfulness meditation intervention using the *10% Happier* app (*practitioners* group) or to a wait-list (*control* group). Study personnel were blinded to group randomization, except for 1 researcher who corresponded with the participants and was not involved in data collection or analysis.

PA students randomized to the practitioner group were asked to practice app-guided mindfulness meditation for approximately 12 minutes per day for 8 weeks. Before randomization and again after completion of the 8-week program, all participants completed self-report assessments and functional magnetic resonance imaging (fMRI), both described in detail below. A total of 2 participants were removed from the analysis for excessive movement during fMRI, resulting in a cohort of 50% (7/14) practitioners (6/7, 86% women) and 50% (7/14) controls (5/7, 71% women; Table 1). Participants were compensated US $100 for completing both assessments. This study was part of a larger preregistered clinical trial (NCT03452670) that included the planned enrollment of several trainee and employee populations. For the larger trial, self-reported perceived incivility was the primary outcome measure and burnout, depression, and anxiety were secondary outcome measures. Enrollment for the larger study did not meet the planned recruitment goals, and this was an exploratory analysis of a subset of the enrolled participants. In addition to the secondary outcome measures, we included a measure of sleep impairment given the prevalence of sleep dysfunction among health profession trainees and the relationship between sleep dysfunction and depression [26].

**Table 1:** Demographic information (N=14).

Characteristics		App, n (%; n=7)	Wait-list, n (%; n=7)	Chi-square (*df*)	*P* value
**Gender**					
	Female	6 (86)	5 (71)	0.4 (1, 14)	.52
	Male	1 (14)	2 (29)	0.4 (1, 14)	.52
**Relationship status**					
	Single	1 (14)	3 (43)	5.7 (3, 14)	.23
	Divorced	0 (0)	1 (14)	5.7 (3, 14)	.23
	In a relationship	4 (57)	2 (29)	5.7 (3, 14)	.23
	Married	2 (29)	1 (14)	5.7 (3, 14)	.23
**Race**					
	White	6 (86)	5 (71)	3.0 (3, 14)	.21
	African American or Black individual	1 (14)	0	3.0 (3, 14)	.21
	Asian	0 (0)	2 (29)	3.0 (3, 14)	.21
	Other	0 (0)	0 (0)	3.0 (3, 14)	.21

### Mindfulness Meditation Intervention

Participants randomized to the meditation group were provided with a 1-year subscription to the app *10% Happier* [27,28]. We chose *10% Happier* among a variety of meditation apps currently in the market given its marketing toward *fidgety skeptics* and its orientation toward practical applications of mindfulness [27], which we thought would appeal to PA students. For example, the app notes, “Just in case you’re worried, meditation does not require a lot of the things people think it might. For example, you don’t have to sit in a particular position. (Unless you want to, of course). You also don’t have to light incense, chant, or believe in anything in particular. There’s nothing to join, no special outfits to wear” [29]. *10% Happier* provided subscriptions for the study participants, suggestions on recommended content, and anonymized app use data. App use was acquired as the elapsed time (in seconds) that the app meditation modules were used by each person.

Students randomized to the meditation group were given the following instructions:

We would like for you to try to practice every day for 8 weeks, even if it is only for one minute. Based on mindfulness research and on the suggestions of the app developers, we would like you to try the following programs: “The Basics” and “Emotional Agility.” If you are pressed for time and cannot do a module from these programs, please do the “One minute counts.”

The *Basics* program included 16 modules with didactic instructions and mindfulness practice time varying between 4:20 to 13:22 minutes (average meditation length: 9:44 minutes, SD 3:17 minutes), and it serves as an introduction to mindfulness meditation. The practices include mindfulness of the sensations of the breath, mindfulness toward sensations and experiences of the body, and mindfulness toward the contents of the mind. In addition, the meditations encourage the practitioner to use the skill of *mental noting* to label their mental contents. The *Emotional Agility* program included 15 modules with didactic instructions and mindfulness toward mental content (focusing on emotions); meditation practice in the *Emotional Agility* program varied from 11:00 to 13:00 minutes (average meditation length: 12:18 minutes, SD 0:40 minutes). The meditation practices in these modules included mindfulness toward the sensations of the body and breath, coupled with other practices aimed at cultivating awareness and understanding of emotions as mental contents and the nonjudgmental stance toward emotions, with a goal of optimizing the response to one’s emotions.

### Self-reported Measures

We measured incivility using the Incivility in Nursing Education—Revised Survey [30], which contains 24 items that ask students about behaviors they exhibited or witnessed in the past 12 months (eg, “students made rude gestures or nonverbal behaviors towards others”). Participants indicated how often these behaviors occurred by selecting from 1=never, 2=rarely (once or twice), 3=sometimes (approximately once per month), or 4=often (more than once per month). Items were summed according to instances of low-level (15 items; eg, “Expressing disinterest, boredom, or apathy about course content or subject matter”) and high-level incivility (9 items; eg, “Making condescending or rude remarks toward others”). Scores were averaged, such that the range was 1-4, with higher scores indicating more incivility exhibited or witnessed.

We measured burnout using the School Burnout Inventory [31], a 9-item survey asking students about how much burnout they have felt in the past month (eg, “I feel overwhelmed by my schoolwork”). Respondents indicated the degree to which they agreed with each statement on a scale of 1-6, where 1=completely disagree and 6=completely agree. Total scores ranged from 9-54, with higher scores indicating more burnout.

The Depression Anxiety and Stress Scale [32] is a 42-item survey asking about feelings of depression, anxiety, and stress that the respondent has experienced in the past week. Participants indicated the degree to which they agreed with each statement on a scale of 0-3, where 0=does not apply to me at all and 3=applied to me very much or most of the time. Each of the three subscales included 14 items. The depression subscale assessed general dysphoria, anhedonia, self-contempt, and hopelessness. A score of 0-9 indicated no depression, 10-13 indicated mild depression, 14-20 indicated moderate depression, 21-27 indicated severe depression, and scores ≥28 indicated extremely severe depression. The anxiety subscale assessed symptoms and subjective feelings related to acute autonomic arousal. A score of 0-7 indicated normal levels of anxiety, 8-9 indicates mild anxiety, 10-14 indicated moderate anxiety, 15-19 indicated severe anxiety, and scores ≥20 indicated extremely severe anxiety.

Sleep dysfunction was measured using the 8-item PROMIS (Patient-Reported Outcomes Measurement Information System) sleep-related impairment (SI) short form 8b, which assessed the frequency with which participants experienced alertness, sleepiness, tiredness, and functional impairments associated with sleep problems during waking hours (eg, “I had difficulty falling asleep”) in the previous 7 days [33]. Items were scored on a 5-point scale, such that higher scores indicated more sleep impairment. Raw scores for each of the eight items were totaled and converted to standardized scores using conversion tables published on the PROMIS website [34].

To assess safety, participants were asked to report their agreement with a number of statements reflecting positive (eg, “I enjoyed using the app”) and negative experiences (eg, “I found it very difficult to do the meditations”) with the app. In addition, they were asked to report anything else *good, bad, or neutral* that they wanted us to know about the app.

### Resting fMRI Image Preprocessing

Baseline and postprogram resting-state fMRI (rsfMRI) data were acquired on a 3T Siemens Prisma FIT (Siemens Healthineers; 8-minute multiband acquisition with 2 seconds repetition time, 2.97×2.97×2.00 mm voxels, 70° flip angle, and echo train length 37). All preprocessing and bivariate correlation (connectome) analyses were performed in the CONN Toolbox (v19c) under MATLAB (vR2019a) [35]. Standard preprocessing methods were applied to the rsfMRI and anatomical volumes in CONN, which wraps SPM8 [36,37] and aCompCor [38] noise source removal functions. It comprised slice timing, field map, and motion correction; coregistration and normalization between rsfMRI images, anatomical images, and Montreal Neurological Institute, standard stereotactic space; smoothing at a 5 mm filter width at half-maximum, which limits intersubject differences and increases signal-to-noise ratio), linear detrending, bandpass filtering at 8-90 mHz; and regression of the 6 motion parameters and their first-order derivatives, along with cerebrospinal fluid and white-matter signals [39-41], by a general linear model (GLM). Scans (repetition time intervals [TRs]) that exhibited motion or global signal change beyond a SD 1.5 IQR limit were marked as *invalid scans* and nulled for the purposes of the GLM (see the Quality Assurance and Quality Control document, Multimedia Appendix 1). Three subjects exhibited 1-8 TRs, out of 240 total TRs, with global signal change beyond this tolerance. These TRs were tagged as *invalid* and were not included in further analyses. We did not add the mean whole-brain signal as a regressor, as there is some evidence that doing so may artificially introduce negative correlations and that the aCompCor method, in combination with bandpass filtering and orthogonalization of motion parameters, is preferable to global signal regression [42]. All structural and *denoized* functional data, gray matter, white matter, and CSF masks were manually inspected to confirm registration validity. In addition, a Fisher (inverse hyperbolic tangent) transformation was applied to bivariate correlation measures before between-subjects analysis to ensure that the connectivity data conformed to the normality assumptions of the GLM (see the Quality Assurance and Quality Control document in the Multimedia Appendix 1).

### Identification of Regions of Interest and Computation of Connectivity Matrices

Regions of interest (ROIs) were computed from preprocessed rsfMRI data using multivariate pattern analysis (Norman et al [43]). The first step of the multivariate pattern analysis procedure, as implemented [31] in the CONN Toolbox, comprised dimensionality reduction into 64 components by singular value decomposition for each subject and condition (group and visit). Subject and condition-specific correlation maps—of size (*number of subjects × number of conditions × number of voxels in each data set*) and comprising standardized (Fisher transformed) bivariate correlation coefficients between each pair of voxels—were then generated from each subject’s reduced-dimensionality data set. Next, a set of four principal components, capturing approximately 95% of the between-subjects or between-conditions variance, was obtained from the aggregate matrix (ie, with all subjects and conditions combined or *stacked*). Finally, the first two of these four principal components were selected for further analysis and subjected to a standard two-way analysis of variance (ANOVA; *group × visit*) to determine whether the components modulated with either condition. Application of a statistical threshold based on Gaussian random field theory [44-46], which estimates error fields within an fMRI statistical map after smoothing with the FWHM kernel (described above), yielded a map of 87 clusters that ostensibly represented any between-group or between-visit differences in voxel-to-voxel functional connectivity across the brain with a cluster growth threshold of *P*≤.05 (uncorrected) and a topological false discovery rate (FDR [47]) of *P*_FDR_≤.05. A total of 16 of these clusters were excluded because of their localization in white matter. Another 15 ROIs representing components of the default mode, sensorimotor, visual, salience, dorsal attention, frontoparietal, language, and cerebellar networks, predefined in the CONN Toolbox, were also included, for a total of 102 ROIs (Table 2 and Figure S1 in Multimedia Appendix 2).

To facilitate testing of our hypothesized relationships among meditation practice time, changes in mental health outcomes, and brain network connectivity, mean signals were extracted from the 71 a posteriori and 15 a priori ROIs for each subject and condition and aggregated into baseline (visit 1) and eighth-week (visit 2 follow-up) ROI-to-ROI connectivity matrices, each of size (*number of subjects × number of ROIs × number of ROIs*). As with the voxel-wise correlation maps, these ROI-ROI connectivity matrices comprised standardized (Fisher transformed) bivariate correlation coefficients between each pair of ROIs. To simplify the within-group analysis, a *delta matrix*, Δconn, was also computed as Δconn=conn_8wks_–conn_baseline_. The baseline, eighth-week, and delta ROI-ROI connectivity matrices (Figure S2 in Multimedia Appendix 2) were leveraged in lieu of voxel-to-voxel connectivity matrices for all further analyses.

**Table 2:** List of the 71 gray matter regions of interest derived from multivariate pattern analysis, with predefined atlas-based regions of interest. Coordinates for region of interest centers of mass and peak voxels (where applicable) are provided in MNI152 standard coordinate space.

Type and CLUSTER_ID		Voxels	Center of mass (MNI^a^)			Peak (MNI)				ATLAS ROI^b^
			x	y	z	x	y	z		
**Brain stem**										
	bs1_bstem	33	–1.2	–29.3	–45.6	0	–30	–46	Brainstem gray matter	
**Basal ganglia**										
	caud1_Rput	126	20.1	27.7	4.8	24	18	4	R putamen/globus pallidus	
	caud2_Rput1	108	29.3	7.1	1.8	34	4	6	R middle insula/putamen	
	caud3_Rgp	45	22.3	–9.4	–3.1	22	–14	–4	R globus pallidus/thalamus/motor thalamus	
	caud4_Rput	36	31.3	–18.7	1.9	32	–16	0	R putamen	
**Cerebellar**										
	cb1_Rlob9	364	4.2	–61.5	–45.7	12	–62	–54	R cerebellum (VIII) lobule IX	
	cb2_Llob6	269	–21.7	–49.8	–46.7	–28	–48	–38	L cerebellum (VI)/prob. WM	
	cb3_Rlob8b	99	16.4	–46.9	–50.3	18	–46	–50	R cerebellum (IX) lobule VIIIb	
	cb4_Rlob7b	82	32	–74.9	–54.1	28	–76	–54	R cerebellum (VII) lobule VIIb	
	cb5_Rcrus2	52	10.5	–72.2	–24.7	6	–72	–28	R cerebellar crus 2/vermis	
	cb6_Lcrus2	39	–48.7	–56.8	–42.6	–56	–56	–44	L cerebellar crus 2/lobule VIIa crus I	
**Cingulate**										
	cing1_Rpcc	426	11	–47.4	20.8	10	–48	18	R precuneus/posterior cingulate/BA23v	
	cing2_Rmicg	21	18.3	–33.3	49.8	18	–34	50	R middle cingulate cortex/ventromedial BA5	
**Frontal**										
	f1_Lba9	408	–3.1	48.3	45	–12	54	42	L superior frontal gyrus/mid BA9	
	f10_Lba6r	33	–50.9	4.3	16.5	–52	4	16	L precentral gyrus/BA44/rostral BA6/caudoventrolateral BA6	
	f11_Rba6ba8	28	18.9	31.7	60.1	20	32	62	R superior frontal gyrus/superior BA6-BA8 transitional area	
	f12_Lba6ba8	27	–31.1	4	59.8	–30	4	60	L middle frontal gyrus/inferior BA6-BA8 transitional area	
	f2_Rba10p	271	18.1	57.5	–13.3	24	62	2	R posterior BA10/superior frontal gyrus	
	f3_Rba8v	207	47.6	10.6	44.5	48	12	48	R ventral BA8A/caudal middle frontal gyrus/IFJ	
	f4_Rba8dl	163	28.7	18.5	49.2	28	20	54	R middle frontal gyrus/dorsolateral BA8A	
	f5_Lba8dl	133	–20	30.5	50.2	–20	30	48	L dorsolateral BA8A/superior frontal gyrus	
	f6_Lsma	124	–15.2	7.9	53.7	–10	12	50	L SMA/supplementary and cingulate eye area/medial frontal gyrus	
	f7_Rba24d	90	1.9	–24.6	46.1	2	–22	50	R dorsal BA24/SMA/superiomedial BA4	
	f8_Rba10d	88	–17.5	62.6	7.9	–22	60	10	L superior frontal gyrus/dorsal BA10/BA46/area Fp1	
	f9_Lba44v	81	–53.4	17.9	2.8	–54	18	4	L ventral BA44/inferior frontal gyrus pars triangularis	
**Medial temporal**										
	mtl1_Lphg1	137	–15.7	–46.5	–10.4	–16	–42	–10	L parahippocampal area 1/subiculum/lingual gyrus/area TH	
	mtl2_Rphg	89	27	–35.9	–13.4	28	–44	–6	R parahippocampal gyrus/rostral lingual gyrus/ventromedial visual area 1	
	mtl3_Renrc	83	21.2	–26.6	–28.5	22	–28	–26	R entorhinal cortex/presubiculum/caudal BA35/36	
	mtl4_Rprc	50	29.6	–1.1	–34.4	30	–2	–34	R perirhinal/ectorhinal cortex/rostral BA36/BA35/parahippocampal gyrus	
	mtl5_Lamg	34	–25.4	–4.1	–21	–24	–4	–16	L piriform cortex/amygdala/laterobasal amygdala	
	mtl6_Lenrc	33	–19.2	–9.4	–33.7	–22	–8	–34	L entorhinal cortex/BA28/BA34/parahippocampal gyrus	
	mtl7_Rhc	23	30.3	–16.2	–18	30	–16	–14	R hippocampus/CA3 fields/CA3 fields	
**Occipital/visual**										
	o1_Rba39rd	287	34.7	–69.8	41.6	36	–72	38	R intraparietal area1/rostro-dorsal BA39/middle occipital gyrus	
	o2_Llop	208	–16.2	–101.6	–2.1	–14	–106	–8	L lateral occipital pole	
	o3_Rv2v3d	29	23.5	–98.9	10.7	24	–98	10	R superior occipital gyrus/visual area V2/dorsal visual area V3	
	o4_Lv2	25	–24.9	–50.5	0	–24	–50	0	L precuneus/prostriate area/lingual gyrus/visual area V2	
	o5_Rba37mv	20	22.3	–42.3	–17.2	22	–44	–18	R ventral visual complex/area FG3/medioventral BA37	
**Opercular**										
	op1_Rro	60	53.9	2.4	2.9	54	4	6	R rolandic operculum/rostral BA6/BA43	
**Orbitofrontal**										
	orb1_Rba11	208	18.7	18.5	–22.8	20	30	–16	R superior orbital gyrus/lateral BA11/area Fo3	
	orb2_Lba10r14m	94	–5.3	41.3	–12.7	–6	44	–16	L rostral BA10/medial BA14/gyrus rectus	
	orb3_Rifgpo	89	32.2	22.9	–31.4	32	22	–26	R inferior frontal gyrus pars orbitalis/superior BA47/lateral BA11/area Fo3	
	orb4_L47l	76	–49.5	29.6	–5.9	–46	28	–6	L lateral BA47/inferior frontal gyrus pars orbitalis	
	orb5_Rba10v	49	4.2	59.6	–11.3	6	60	–10	R ventral BA10/medial BA11/middle orbital gyrus	
	orb6_Lpofc	36	–14	14.1	–14.4	–14	14	–14	L gyrus rectus/area Fo2/posterior orbitofrontal complex/subcallosal gyrus	
	orb7_Lba11l	34	–20.1	32.1	–18.8	–18	34	–22	L superior orbital gyrus/area Fo3/lateral BA11	
	orb8_Rofc	25	2.1	16.1	–21.7	2	16	–22	R orbitofrontal cortex/area Fo2	
	orb9_Rba14m	20	4.6	28.5	–13.2	4	30	–14	R middle orbital gyrus/area s32/area 25/medial BA14	
**Prefrontal**										
	pfc1_Lba9pv	50	–37.5	33.8	22	–40	28	26	L posterior ventral BA9/BA46	
	pfc2_Lba9av	40	–38.1	55.2	1.6	–38	54	0	L anteroventral BA9/46/rostral BA47	
**Parietal**										
	pl1_Lipl	280	–35	–67.6	25.1	–36	–68	24	L middle occipital gyrus/inferior parietal lobule/rostro-ventral BA39	
	pl2_Lba7m	233	–2.9	–53.5	58.1	–6	–52	66	L medial BA7A/medial BA5	
	pl3_Lba7m5ml	190	44.6	20.4	10.3	46	16	6	L medial BA7A/mediolateral BA5	
	pl4_Lpos2	114	–4.2	–65.9	42.6	–2	–70	44	L parieto-occipital sulcus area 2/medial BA7/precuneus	
	pl5_Rpos2	32	16.9	–59.5	28.2	20	–60	26	R precuneus/parieto-occipital sulcus area 2	
**Temporal**										
	t1_Lffc	95	–46.6	–56.9	–18.9	–44	–60	–16	L fusiform gyrus/fusiform face complex/lateroventral BA37/area FG2	
	t10_Ravsts	31	56.3	–7.2	–21.6	56	–6	–20	R anteroventral superior temporal sulcus/middle temporal gyrus	
	t11_Laud5	31	–61.5	–0.3	–8.7	–64	–4	–4	L superior temporal gyrus/area TE3/auditory 5 complex	
	t12_Rba38l	28	47.2	21.4	–26.3	48	22	–26	R temporopolar cortex/lateral BA38/dorsal area TG	
	t13_Lpiri	6	–41.7	6	–10.7	–40	4	–12	CLUSTER5/L piriform cortex/insula/posterior insula area 2	
	t14_Lba37vl	3	–48.7	–64	–3.3	–48	–64	–4	CLUSTER10/middle temporal gyrus/area PH/ventrolateral BA37	
	t2_Lba38l	68	–35.3	4	–23.2	–38	6	–20	L dorsal area TG/lateral BA38/temporopolar cortex	
	t3_Rba22	64	66.9	–17.3	0	66	–14	0	R auditory 4 complex/area TE3/caudal BA22	
	t4_Lba20il	62	–54.8	–11.9	–34.2	–56	–12	–32	L anterior area TE2/inferolateral BA20/inferior temporal gyrus	
	t5_Lba22c	60	–65.2	–46.7	11.8	–68	–48	12	L superior temporal visual area/caudal BA22	
	t6_Rins	45	34.2	–16.7	23.4	34	–14	22	R insula/area OP2-3/VS	
	t7_Lpins2	37	–35.4	–6.4	–6.5	–36	–8	–4	L posterior insular area 2/circular insula	
	t8_Lba38m	36	–45.8	24	–25.4	–44	26	–26	L temporopolar cortex/dorsal area TG/medial BA38	
	t9_Raud5	33	65.1	–38.6	5.5	66	–38	6	R middle temporal gyrus/auditory 5 complex/ventral superior temporal sulcus	
**Thalamic**										
	th1_Lpfthal	82	–5.7	–14.4	–3.5	–6	–14	–2	L thalamus/thalamic area IPF/prefrontal-directed thalamus	
	th2_Rpmthal	72	15.5	–18.6	4.9	16	–18	6	R thalamus/premotor-directed thalamus	
**Predefined**										
	medial PFC^c^ (DMN^d^)	—^e^	1	55	–3	N/A^f^	N/A	N/A	medial prefrontal cortex, default mode network	
	L lateral parietal (DMN)	—	–39	–77	33	N/A	N/A	N/A	L lateral parietal cortex/von Economo PG, default mode network	
	R lateral parietal (DMN)	—	47	–67	29	N/A	N/A	N/A	R lateral parietal cortex/von Economo PG, default mode network	
	posterior cingulate (DMN)	—	1	–61	38	N/A	N/A	N/A	posterior cingulate cortex, default mode network	
	L lateral sensorimotor	—	–55	–12	29	N/A	N/A	N/A	L lateral sensorimotor cortex	
	R lateral sensorimotor	—	56	–10	29	N/A	N/A	N/A	R lateral sensorimotor cortex	
	superior sensorimotor	—	0	–31	67	N/A	N/A	N/A	superior (mesial) sensorimotor cortex	
	medial visual	—	2	–79	12	N/A	N/A	N/A	medial visual cortex/Brodmann 18/calcarine gyrus/visual area V1	
	occipitopolar visual	—	0	–93	–4	N/A	N/A	N/A	occipitopolar visual cortex/Brodmann 17/calcarine gyrus	
	L lateral visual	—	–37	–79	10	N/A	N/A	N/A	L lateral visual cortex/visual area V4	
	R lateral visual	—	38	–72	13	N/A	N/A	N/A	R lateral visual cortex/visual area V4	
	anterior cingulate (SN^g^)	—	0	22	35	N/A	N/A	N/A	anterior cingulate cortex, salience network	
	L anterior insula (SN)	—	–44	13	1	N/A	N/A	N/A	L anterior insula, salience network	
	R anterior insula (SN)	—	47	14	0	N/A	N/A	N/A	R anterior insula, salience network	
	L rostral PFC (SN)	—	–32	45	27	N/A	N/A	N/A	L rostral prefrontal cortex, salience network	
	R rostral PFC (SN)	—	32	46	27	N/A	N/A	N/A	R rostral prefrontal cortex, salience network	
	L supramarginal g. (SN)	—	–60	–39	31	N/A	N/A	N/A	L supramarginal gyrus/Brodmann 40, salience network	
	R supramarginal g. (SN)	—	62	–35	32	N/A	N/A	N/A	R supramarginal gyrus/Brodmann 40, salience network	
	L FEF (DAN^h^)	—	–27	–9	64	N/A	N/A	N/A	L frontal eye fields, dorsal attention network	
	R FEF (DAN)	—	30	–6	64	N/A	N/A	N/A	R frontal eye fields, dorsal attention network	
	L inferior parietal s. (DAN)	—	–39	–43	52	N/A	N/A	N/A	L inferior parietal sulcus, dorsal attention network	
	R inferior parietal s. (DAN)	—	39	–42	54	N/A	N/A	N/A	R inferior parietal sulcus, dorsal attention network	
	L lateral PFC (FPN^i^)	—	–43	33	28	N/A	N/A	N/A	L lateral prefrontal cortex/Brodmann 9/46, frontopolar network	
	L posterior parietal cortex (FPN)	—	–46	–58	49	N/A	N/A	N/A	L posterior parietal cortex/von Economo PFm, frontopolar network	
	R lateral PFC (FPN)	—	41	38	30	N/A	N/A	N/A	R lateral prefrontal cortex/Brodmann 9/46, frontopolar network	
	R posterior parietal cortex (FPN)	—	52	–52	45	N/A	N/A	N/A	R posterior parietal cortex/von Economo PFm, frontopolar network	
	L inferior frontal language area	—	–51	26	2	N/A	N/A	N/A	L inferior frontal gyrus	
	R inferior frontal language area	—	54	28	1	N/A	N/A	N/A	R inferior frontal gyrus	
	L posterior STG^j^ language area	—	–57	–47	15	N/A	N/A	N/A	L posterior superior temporal gyrus	
	R posterior STG language area	—	59	–42	13	N/A	N/A	N/A	R posterior superior temporal gyrus	
	Anterior cerebellum	—	0	–63	–30	N/A	N/A	N/A	Anterior cerebellum	
	Posterior cerebellum	—	0	–79	–32	N/A	N/A	N/A	Posterior cerebellum	

^a^MNI: Montreal Neurological Institute stereotactic coordinate system.

^b^ROI: regions of interest.

^c^PFC: prefrontal cortex.

^d^DMN: default mode network.

^e^Not available (for predefined ROIs, subject signals for correlation analyses were derived from the average signal over the entire region and cluster sizes are not available).

^f^N/A: not applicable (for predefined ROIs, subject signals for correlation analyses were derived from the average signal over the entire region and peak voxel locations are not available).

^g^SN: salience network.

^h^DAN: dorsal attention network.

^i^FPN: frontopolar network.

^j^STG: superior temporal gyrus.

### Statistical Analysis

Descriptive statistics (means, SDs, and SEs) were used to characterize baseline demographics and responses to pre- and postintervention psychometric assessments. Meditation practice time was the sum of the 8 weeks of practice, which was provided by the app company. Missing items in the psychometric scales were estimated with expectation maximization [48] (missing items never accounted for >5% of the total data) using other items within the scale as predictor variables. Baseline differences between the app and wait-list groups were assessed using independent *t* tests for continuous variables and chi-square tests for categorical variables (Tables 1 and 3). Post- versus preintervention differences in burnout, depression, anxiety, or sleep impairment in the *mindfulness* group were assessed by repeated-measures ANOVA. Given the exploratory nature of our analyses, we first used an α level of .05. Tests of the hypotheses were also conducted using Bonferroni-adjusted α levels of .008 (.05/6 outcome variables). To evaluate whether statistically significant outcomes were related to mindfulness practice time, we conducted Spearman correlation analyses between practice time and changes in all relevant outcomes.

**Table 3:** Group means and differences with respect to the Patient-Reported Outcomes Measurement Information System sleep-related impairment short form 8b (sleep disturbance), School Burnout Inventory (burnout), and The Depression Anxiety and Stress Scale (depression and anxiety) scores at baseline and >8 weeks. Sleep disturbance indices were lower in the app group than the wait-list group at >8 weeks.

	Time 1				Time 2				*F* test (*df*)
	App group, mean (SD)	Wait-list group, mean (SD)	*t* test (*df*)	App group, mean (SD)		Wait-list group, mean (SD)	*t* test (*df*)		
Sleep disturbance	51.9 (9.99)	54.1 (2.99)	–0.57 (12)	42.2 (7.45)		53.4 (7.15)	–2.93^a^ (12)	8.68^a^ (1, 12)	
Depression	3.00 (2.23)	4.86 (2.67)	–1.41 (12)	3.14 (2.41)		4.00 (2.83)	–0.61 (12)	0.48 (1, 12)	
Anxiety	5.14 (3.08)	8.00 (3.96)	–1.51 (12)	3.43 (2.82)		4.57 (4.20)	–0.60 (12)	0.28 (1, 12)	
Burnout	30.7 (6.21)	34.0 (5.48)	–1.05 (12)	29.9 (8.69)		34.9 (7.67)	–1.14 (12)	0.58 (1, 12)	

^a^Statistically significant at *P*<.05.

### Differences in Connectivity by Group, Visit, and Practice Time Bin

All within- and between-group statistical analyses were performed in MATLAB v2019a. Within-group differences in connectivity distributions (mindfulness at 8 weeks vs mindfulness at baseline and wait-list at 8 weeks vs wait-list at baseline) were assessed against each ROI pair in the baseline and eighth-week ROI-ROI connectivity matrix, as appropriate, using a 2-tailed *t* test assuming equal variances (MATLAB ttest2() function). Correction for FDR (the expected proportion of false discoveries between all ROI-ROI pairs with similar or larger effects) was performed at a critical value of α=.05 via the Benjamini-Hochberg procedure (MATLAB mafdr() function [49]). Similarly, between-group differences in connectivity (mindfulness at 8 weeks vs mindfulness at baseline and wait-list at 8 weeks vs wait-list at baseline) were assessed against each ROI pair in the Δconn matrix via one-way ANOVA omnibus test (anova1() function) using *group* as a predictor, with post hoc comparisons by 2-tailed *t* test. In addition, mindfulness subjects were divided into low (practice time <53 minutes), moderate (53 to <225 minutes), and high (≥225 minutes) practice time bins, and a 1-way ANOVA was conducted against the Δconn matrix using *practice time bin* as the predictor and post hoc comparisons by 2-tailed *t* tests. In each of the above cases, *P* values were FDR corrected using the Benjamini-Hochberg procedure at α=.05.

### Differences in Connectivity by Practice Time, Mental Health Scores, and Mental Health Scores

For any mental health outcomes that had a significant group-by-time interaction, we examined whether the change or changes were related to changes in functional connectivity. As our analyses indicated that the only significant impact of app-delivered mindfulness was on the PROMIS sleep impairment measure, we limited our analyses to this measure. Although connectivity data were normally distributed because of the application of the Fisher transform in preprocessing, the ordinal nature of PROMIS sleep impairment scores across all subjects, skewness of the practice time data within the mindfulness group, and the presence of outliers in SI and practice time data rendered parametric approaches inappropriate for analysis of these predictors relative to connectivity. Consequently, we used a rank-based method, Spearman rho, to investigate such relationships. Spearman rank-order correlations (ρ) are equivalent to the Pearson product-moment correlation coefficient but are applied to ranks rather than continuous values and are, therefore, less susceptible to strong outliers. Spearman correlations were calculated for practice time (mindfulness subjects only) and change in SI score (ΔSI=SI_8wks_–SI_baseline_) versus Δconn using the MATLAB function corr(). FDR correction was performed as previously described, except that *P* value rankings were computed over all ROI-ROI pairs *within the mindfulness group* only for ρ (practice time and Δconn) analysis. All *P* values were FDR corrected, as previously described.

### Machine Learning Classifiers for Sleep Impairment Based on Connectivity

Of the 7 subjects randomly assigned to the mindfulness group, 3 (43%) reported sleep impairment at baseline but no longer reported impairment at 8 weeks, whereas, of the 7 waitlisted subjects, 3 (43%) had sleep impairment at baseline and continued to exhibit impairment at 8 weeks (Multimedia Appendix 2, Figure S4). Therefore, we were interested in testing whether conversion between sleep impairment and nonimpairment might be predicted by a supervised learning algorithm based on the change in functional connectivity (Δconn) between baseline and >8 weeks. Accordingly, per-visit labels were assigned to each subject based on the PROMIS-derived classification of sleep impairment, such that a PROMIS SI score commensurate with sleep impairment was encoded as 1 and nonimpairment as 0. A ΔIMPAIRMENT score (IMPAIRMENT_8wks_–IMPAIRMENT_baseline_) was then computed and used as target classes (0=*no change*, 11 subjects total; 1=*improvement*, 3 subjects total; and −1=*decline*, 0 subjects). A corpus of six binary classifiers, comprising support vector, gradient boosting, random forest, Gaussian naïve Bayes, linear discriminant, and multilayer perceptron estimators were implemented in *scikit-learn*, v0.22.1, under Python 3.7.7 (see Multimedia Appendix 2 for training and testing details). After excluding undefined values (NaNs) corresponding to the conn diagonal from the matrix, conn samples and their associated labels ϵ {−1, 0, +1} were split into training (30%) and testing (70%) sets. The samples were reshuffled into train/test sets over 5 tuning rounds. In each round, the classifier hyperparameters were tuned by random search (RandomizedSearchCV() in *scikit-learn*) over 5000 iterations.

**Figure 1:**
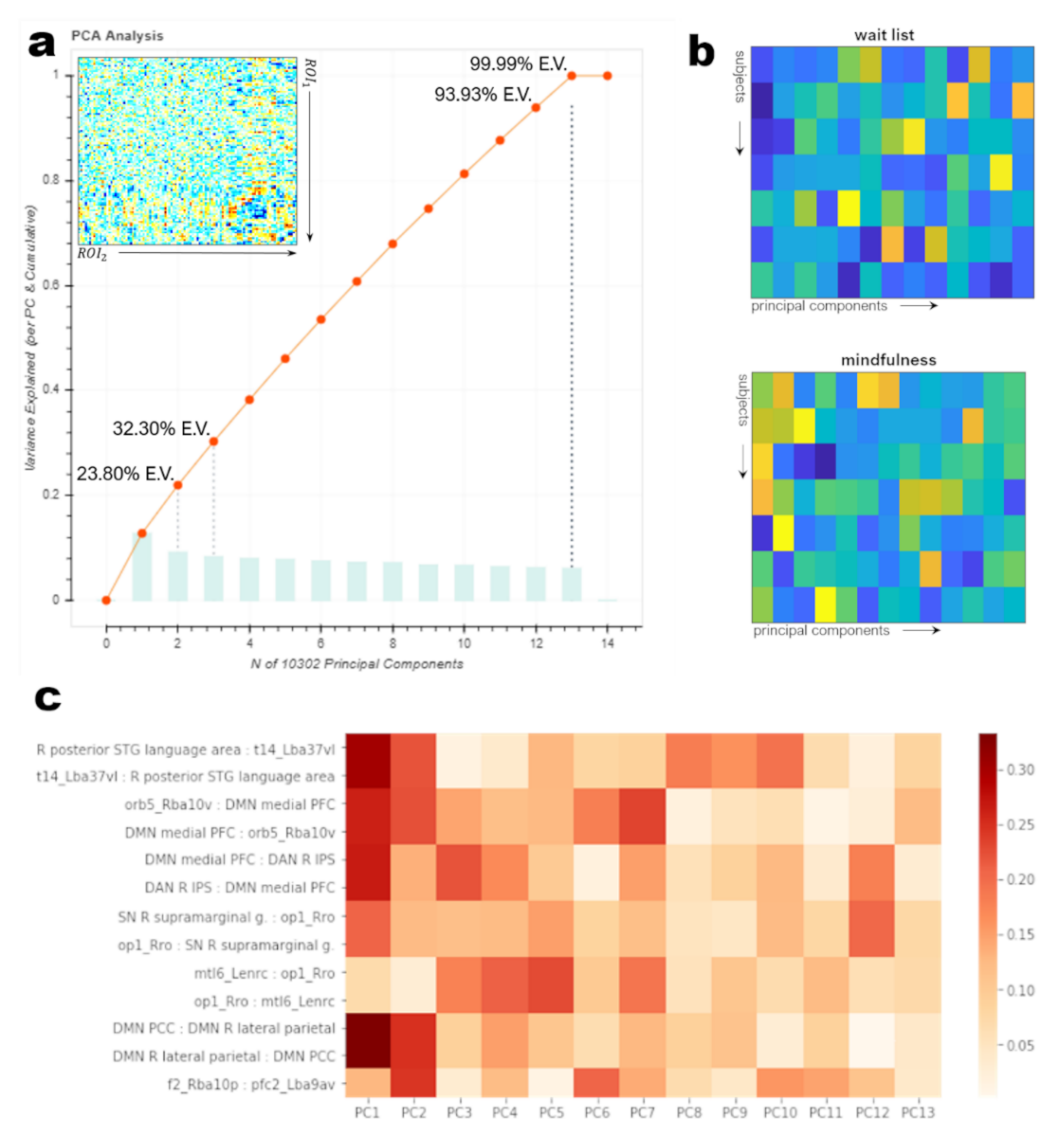
Results of principal components decomposition of Δconn matrix. (a) explained variance and cumulative explained variance for the first 14 principal axes. 13 components accounted for 99.99% of the explained variance in the delta matrix (Δconn), suggesting a large amount of multicollinearity across ROI-ROI connections. The recomposition of the Δconn matrix from the 13 selected components is shown in the inset. (b) within-group correlations between per-subject Δconn values and the 13 selected components, averaged across subjects within each group. (c) Primary contributors to each component based on the dot product of the original Δconn matrix and the 13-PC decomposition. Note the substantive contributions of posterior superior temporal, fusiform (t14), orbitofrontal, and default mode, dorsal attention, and salience-network components to components PC1 and PC2. These two components represented nearly one-third of the explained variance in the Δconn matrix. DAN: dorsal attention network; DMN: default mode network; PFC: prefrontal cortex; SN: salience network; STG: superior temporal gyrus.

A second series of classification runs were performed to test whether further dimensionality reduction improved the classifier performance. This second series leveraged the same classifiers used previously but the *features* (ROI-ROI connections) used for classification comprised 13 principal components—accounting for 99.99% of the data variance—derived from the conn matrix using decomposition. PCA() in *scikit-learn*. The contribution of each feature (connection) to each of the 13 components was ascertained by taking the magnitude of the dot product of the conn matrix, renormalized to the explained variance, and the principal components, yielding a collinearity metric between each connection and component. The results of the PCA decomposition and the connections assigned to each feature are shown in Figure 1, which also shows the contribution of each feature (connection in the conn matrix) to the 13 principal components recovered by computing the dot product between the Δconn matrix and the derived components. Hyperparameters were computed separately for this second series of classifications, following the strategy previously described.

## Results

### Overview

At time 1, before randomization, the groups showed no differences in any self-report variables (all *P* values>.16) or demographic variables (all *P* values>.21). Participants randomized to the mindfulness group used the app between 0 and 466.2 (mean 182.8, SD 182.8) minutes. A total of 2 trainees randomized to the practitioner group did not use the app at all; however, these PA students were included in all analyses in an intent-to-treat design. None of the participants reported adverse events or adverse experiences with the app.

**Figure 2:**
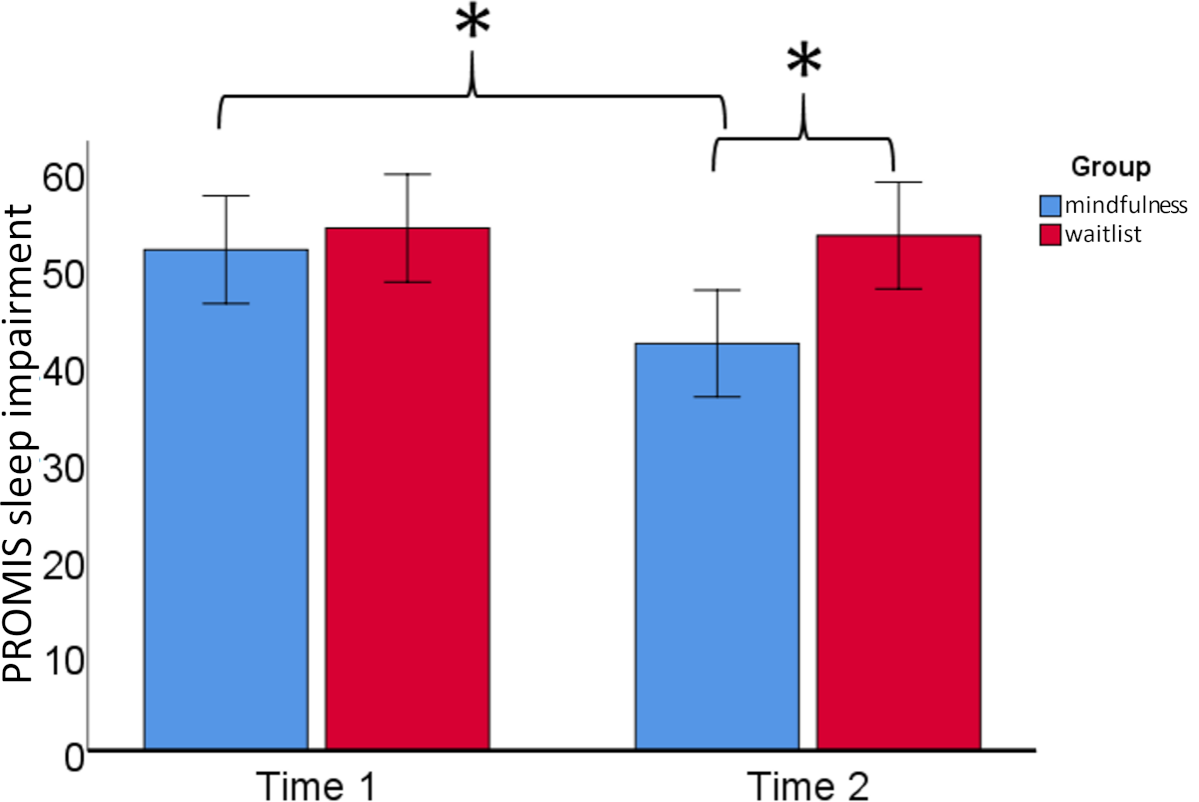
Differences in sleep impairment by group (mindfulness vs waitlist) and visit (time 1, baseline; Time 2, >8 weeks). An asterisk (*) indicates significant differences in sleep impairment at *P*≤.05. PROMIS: Patient-Reported Outcomes Measurement Information System.

There was a significant group-by-time interaction for sleep impairment, such that participants randomized to mindfulness group reported a reduction in impairment compared with those randomized to the wait-list group (*F*_12_=8.68; *P*=.01; η_p_^2^=0.42). No other self-reported outcomes were significant for the group-by-time interaction (low incivility: *F*_12_=0.27; *P*=.61; high incivility: *F*_12_=0.29; *P*=.60; burnout: *F*_12_=0.56; *P*=.47; depression: *F*_12_=0.53; *P*=.48; anxiety: *F*_12_=1.23; *P*=.28). A paired sample *t* test indicated that participants in the mindfulness group reported significant reductions in sleep impairment (*t*_6_=3.35; *P*=.02). Students randomized to the mindfulness group did not report any other significant changes, although there was a trend toward a reduction in burnout (*t*_6_ 2.20; *P*=.07). Paired sample testing indicated that the wait-list control group reported a reduction in anxiety (*t*_6_=3.62; *P*=.01). Finally, independent sample *t* tests indicated that there was a significant difference between the mindfulness and wait-list groups in terms of sleep impairment at time 2 (*t*_12_=–2.93; *P*=.02). There were no other significant differences between the groups at time 2. Finally, changes in sleep impairment were not significantly correlated with mindfulness practice time within the mindfulness group (Spearman *r*_s_=–0.36; *P*=.43; Figure 2).

### Connectivity by Group and Visit

A 2-way ANOVA was conducted to assess whether *group* (mindfulness or control) and/or *visit* (baseline and >8 weeks) were significant predictors of between- and within-group variance in the baseline and eighth-week connectivity matrices or the *delta matrix* (Δconn=connectivity at >8 weeks − connectivity at baseline). ANOVAs were conducted on each ROI-ROI pair separately, and *P* values were corrected for FDR using the Benjamini-Hochberg procedure. We failed to find significant effects of the *group × visit* interaction for any connection, and subsequent inspection of the main effects indicated that although *group* and *visit* each accounted for a statistically significant amount of variance across the connectivity matrices, they did not affect the same connections. Consequently, main-effects ANOVAs and subsequent post hoc analysis by a 2-tailed *t* test were conducted for *group* and *visit* separately.

The ANOVA on *group* conducted against the baseline and eighth-week connectivity matrices indicated significant differences in control subjects’ connectivity among the supplementary motor, middle temporal, inferior temporal, occipitopolar, and orbitofrontal cortices at baseline, and among the insular, cerebellar, lateral visual, and superior frontal cortices and thalamus at >8 weeks (Table 4). Post hoc *t* tests revealed that, at baseline, mindfulness participants exhibited stronger connectivity than controls with respect to left supplementary motor–left ventrolateral Brodmann area 37 (*t*_12_=5.37; *P*_FDR_=.02) and left inferolateral Brodmann area 20–right middle temporal gyrus (*t*_12_=4.73; *P*_FDR_=.05). The left BA37 ROI was localized to the ventrolateral aspect of the ipsilateral middle temporal gyrus corresponding to the area PH of Economo-Koskinas [50], and the left BA20 ROI was localized to the anterior aspect of visual area TE2. In contrast, controls exhibited stronger left lateral occipitopolar–ipsilateral superior temporal gyrus connectivity than the mindfulness subjects (*t*_12_=−4.98; *P*=.03) at baseline. The latter ROI was associated with area TE3 and/or auditory complex 5. At >8 weeks, mindfulness subjects exhibited stronger connectivity than control subjects with respect to the left dorsolateral Brodmann area 8–left posterior insula (*t*_12_=4.84; *P*_FDR_=.05), left cerebellar lobule VI–right lateral visual cortex (*t*_12_=4.65; *P*_FDR_=.05), and right premotor thalamus–right lateral visual cortex (*t*_12_=4.32; *P*_FDR_=.05).

**Table 4:** Results of *t* tests on group (mindfulness, or MF, vs wait-list, or CX) and visit (visit 1, v1, vs visit 2, v2). Connections surviving false discovery rate correction are presented as pairs of source and versus regions of interest.

Group and sources		Versus	*t* test (*df*)^a^	*P*_FDR_
**MF v1>CX v1**				
	f6_Lsma	t14_Lba37vl	5.3665 (12)	.02
	o2_Llop	t11_Laud5	–4.977 (12)	.03
	t4_Lba20il	t9_Raud5	4.7331 (12)	.049
**MF v2>CX v2**				
	f5_Lba8dl	t7_Lpins2	4.7415 (12)	.048
	cb2_Llob6	R lateral visual	4.6543 (12)	.05
	th2_Rpmthal	R lateral visual	4.3193 (12)	.05
**MF v2>MF v1**				
	posterior cing. (DMN)	anterior cing. (SN)	5.7184 (7)	.009
	orb2_Lba10r14m	t7_Lpins2	5.5659 (7)	.01
	f11_Rba6ba8	anterior cingulate (SN)	4.9121 (7)	.02
	R FEF (DAN)	R inf. frontal lang. area	4.8411 (7)	.04
	posterior cing. (DMN)	L anterior insula (SN)	4.1621 (7)	.04
	cing1_Rpcc	L anterior insula (SN)	4.1902 (7)	.04
	orb5_Rba10v	t7_Lpins2	4.1199 (7)	.047
	t7_Lpins2	medial PFC (DMN)	4.6925 (7)	.05
**CX v2>CX v1**				
	o2_Llop	mtl5_Lamg	5.4641 (5)	.01

^a^Two-tailed *t* test.

The ANOVA on *visit* conducted against the delta matrix Δconn indicated that baseline-to-eighth-week connectivity differed between mindfulness and control subjects with respect to connections between the left lateral occipitopolar cortex and the ipsilateral amygdala and in mindfulness subjects’ connectivity among the anterior and posterior cingulate, insular, orbitofrontal, and medial prefrontal cortices (Tables 4 and 5). Post hoc *t* tests subsequently revealed that control subjects exhibited higher connectivity between left lateral occipitopolar cortex and left amygdala at >8 weeks than at baseline (*t*=5.46; *P*_FDR_=.01). In contrast, mindfulness participants exhibited higher connectivity at >8 weeks, relative to baseline, between anterior cingulate (an SN component) and posterior cingulate (a DMN component; *t*=5.72; *P*_FDR_=.01), left rostral orbitofrontal Brodmann area 10 and ipsilateral posterior insula (*t*=5.57; *P*_FDR_=.01), anterior cingulate and right superior frontal gyrus (*t*=4.91; *P*_FDR_=.02), right frontal eye fields (a dorsal attention network component) and ipsilateral inferior frontal gyrus (*t*=4.84; *P*_FDR_=.04), posterior cingulate and left anterior insula (an SN component; *t*=4.16; *P*_FDR_=.04), the ventral aspect of the right orbitofrontal Brodmann area 10 and left posterior insula (*t*=4.12; *P*_FDR_=.05), and left posterior insula and medial prefrontal cortex (*t*=4.69; *P*_FDR_=.05). The superior frontal ROI was associated with an area denoted *i6-8* (Assem et al [51]) located in the superior aspect of the transition area between the premotor cortex (Brodmann area 6) and frontal eye fields (Brodmann area 8; Table 2).

**Table 5:** Results of one-way analysis of variance on delta matrix with group as the predictor. Post hoc analysis by 2-tailed *t* test. *P* values false discovery rate-corrected by Benjamini-Hochberg procedure. Note that, as the connectivity coefficients were normalized, they are directly comparable with a Cohen d statistic and thus indicate effect sizes. The differences listed here fall in the range of 0.15-0.35 and should be regarded as small-to-moderate effects.

ID1	ID2	ROI1	ROI2	*F* test (*df*)	MSE^a^	Model *P* value	Post hoc	Mean difference	*P* value
t1_Lffc	orb2_Lba10r14m	L fusiform face complex	L r. BA10/med. BA14	20.1465 (1, 12)	0.016	<.001	Control<mindfulness	–0.3031	<.001
pl2_Lba7m	orb1_Rba11	L med. BA7/med. BA5	R lat. BA11/area Fo3	15.3067 (1, 12)	0.0285	.002	Control<mindfulness	–0.3531	.002
Anterior cerebellum	Posterior cerebellum	Anterior cerebellum	Posterior cerebellum	8.8866 (1, 12)	0.0179	.01	Control<mindfulness	–0.2134	.01
mtl3_Renrc	cb4_Rlob7b	R entorhinal ctx./presubi	R cerebellar lobule VIIb	6.3201 (1, 12)	0.0162	.03	Control<mindfulness	–0.171	.03
pl4_Lpos2	caud2_Rput1	L parieto-occip. s./medial BA7	R mid. insula/putamen	5.9626 (1, 12)	0.0215	.03	Control<mindfulness	–0.1914	.03
pl3_Lba7m5ml	f4_Rba8d	L med. BA7/med. lat. BA5	R mid. frontal g./BA 8	5.9356 (1, 12)	0.051	.03	Control<mindfulness	–0.294	.03
cb2_Llob6	pl2_Lba7m	L cerebellar lobule VI	L med. BA7/med. BA5	4.9141 (1, 12)	0.018	.046	Control<mindfulness	–0.1588	.046
orb3_Rifgpo	mtl2_Rphg	R IFG orb./sup. BA47/lat. BA11	R parahippocampal g	4.889 (1, 12)	0.0167	.047	Control<mindfulness	–0.1527	.047
mtl1_Lphg1	f5_Lba8dl	L parahippocampal g./subi	L dors. lat. BA8	4.7757 (1, 12)	0.027	.049	Mindfulness<control	0.1919	.049

^a^MSE: mean square error.

### Changes in Connectivity With the SI Score and Practice Time

Nearly all subjects exhibited a decrease in SI scores between baseline (control: 54.1 [SD 2.99]; mindfulness: 51.89 [SD 9.99]) and +8 weeks [control 53.36 [SD 7.15]; mindfulness: 42.19 [SD 7.45]). Mean ΔSI scores, defined as SI score at >8 weeks minus SI score at baseline, were −0.76 (SD 5.13) and −9.70 (SD 6.18) for the control and mindfulness groups, respectively. Spearman rank correlation, assessed against the delta matrix (Δconn, defined as connectivity among the 102 ROIs at >8 weeks minus connectivity at baseline) across all subjects, indicated that a greater decrease in SI score was associated with increased connectivity between the right superior frontal gyrus (BA6/8 transition area, *i6-8*) and ipsilateral inferior parietal sulcus (ρ=0.82, *P*_FDR_=.03) and between the left superior temporal gyrus and medial prefrontal cortex (a component of the DMN; ρ=0.81; *P*_FDR_=.05). The superior temporal ROI was localized to the caudal aspect of Brodmann area 22 (Wernicke area) and the superior temporal visual cortex. Greater decreases in SI score were also associated with reduced connectivity between the left supplementary motor area and the ventrolateral aspect of the ipsilateral middle temporal gyrus/Economo-Koskinas area PH (ρ=0.89; *P*_FDR_<.01; Table 6).

Within the mindfulness group, Spearman correlations indicated an association between practice time and changes in the connectivity of four connections: between the right inferior parietal sulcus (a component of the dorsal attention network) and right lateral visual cortex, between the right inferior parietal sulcus and the occipitopolar visual cortex, between the right middle orbital gyrus and orbitofrontal Brodmann area 11, and between the right middle orbital gyrus and the left lateral sensorimotor cortex. The strengths of these connections increased linearly with practice time, with the exception of the left lateral sensorimotor–middle orbital gyrus connection, which decreased with practice time (Tables S1 and S2 and Figure S3 in Multimedia Appendix 2).

**Table 6:** Spearman correlations (rho) between the connectivity strength of selected connections and change in sleep impairment score (ΔSI, defined as sleep impairment at 8 weeks minus sleep impairment at baseline), taken across all subjects. Correlations are corrected for multiple comparisons using false discovery rate.

Source	Versus	rho	*P*_FDR_
f6_Lsma	t14_Lba37vl	0.8896	.002
t5_Lba22c	DMN medial PFC	–0.8057	.05
f11_Rba6ba8	DAN R IPS	–0.8234	.03

### Classifiers

Throughout 5 rounds of training or test group shuffling, each with 5000 iterations of hyperparameter tuning, the 6 estimators (support vector, gradient boosting, random forest, Gaussian naïve Bayes, linear discriminant, and multilayer perceptron) were equally proficient in classifying the change from sleep impairment at baseline to no sleep impairment at >8 weeks based on the full 102 × 102-ROI delta matrix (Δconn) for each subject. All estimators correctly classified true converters; conversely, they misclassified at least 1 nonconverter in each of the 5 rounds. Reducing the dimensionality of the Δconn matrix to 13 components (99.99% of the cumulative explained variance; Figure 1) via principal component analysis increased the accuracy of most classifiers by 3-5% by increasing estimator specificity (decreasing the false positive rate). Conversely, the estimators tended to misclassify true converters as nonconverters, increasing the false-negative rate, when predictions were based on the reduced-dimensionality delta matrix. Under this schema, Gaussian naïve Bayes and random forest classifiers slightly outperformed other estimators, with an accuracy rate of approximately 80%. We must, however, urge caution in overinterpreting these results, given the small number of true converters (n=3) to nonconverters (n=11) and the absence of subjects *converting* from nonsleep-impaired to sleep-impaired.

## Discussion

### Principal Findings

Provider burnout and depression have profound national and institutional economic costs, as well as deep societal and social effects. Burnout costs an estimated US $150 billion per year or almost 5% of the nation’s health care expenditure [52]. Although these costs are generally estimated based on the effects of burnout and depression among physicians, PAs also report remarkably high levels of burnout [5] and depression [6]. Although little is known about the public health impact of PA burnout, physician burnout increases malpractice rates, exacerbates physician shortages, erodes both health care organization morale and patient experience [53], and reduces clinical effectiveness [54,55]. Given their overlapping roles and day-to-day activities, it is likely that burnout has a comparable effect on PAs.

Similar to the relative lack of data on burnout and well-being among practicing PAs, far less is known about the mental health needs of PA students than is known about medical residents and medical students. The PA profession is in a period of some flux, with a workforce population that is growing in overall number and that is increasingly young and female [4,56]. PA students may have unique needs, given the differences between PAs and physicians. PA students often report choosing their professional route based on concerns about debt load [57] and expectations of a healthier work-life balance [58]. Here, students reported low levels of depression (0% of students) and relatively low levels of anxiety. Approximately 21% (3/14) of students indicated moderate anxiety, and 7% (1/14) of students indicated severe anxiety at the outset of the study. More students (43%) reported sleep impairment; 29% (4/14) of students reported mild impairment, and 14% (2/14) of students reported moderate impairment before randomization. It is important to note that the students who enrolled in the study may not be representative of the entire PA student population.

Mindfulness meditation has shown great promise for improving sleep disruption and insomnia symptoms [13,14], and it may be beneficial to health professional trainees who often experience high rates of sleep dysfunction [16]. However, most studies examining mindfulness among health professionals and trainees have examined time-intensive interventions that are prohibitive for many. Previous studies have shown that app-delivered mindfulness may be effective in reducing anxiety among physicians [59]. Although we included a wait-list control group to control self-selection and the inevitable changes that occur during PA school but not attributable to the intervention, future studies should include an active control condition. Although this has been challenging in studies of app-delivered mindfulness to date, recent work has advanced in this area toward developing smartphone apps that can be used as active comparators (eg, Huberty et al [60]).

PA students randomized to mindfulness reported a significant reduction in sleep impairment compared with students randomized to the wait-list. Although 43% (3/7) of students randomized to mindfulness reported mild (*t*=55-59.9) or moderate (*t*=60-69.9) sleep impairment before randomization, none of the students randomized to mindfulness reported sleep impairment after training (*t*<55) [61]. These findings are consistent with previous research indicating that mindfulness-based interventions are effective in reducing sleep disturbance and for altering neurobiology related to the default mode in adults with sleep disturbances [62]. Our findings extend previous work to indicate that short, app-delivered mindfulness is beneficial for improving self-reported sleep impairment. A recent study found that most study participants using a popular mindfulness app (Calm) downloaded it to improve sleep impairment [63]; considering our data further highlights the potential importance of app-delivered mindfulness in the context of sleep and sleep dysfunction. This study also adds to what is known about the impact of mindfulness on medical trainees [64] and may be part of a critical solution for sleep dysfunction, which is associated with an increased risk of depression, burnout, and medical errors [65,66]. Although changes in sleep impairment were not correlated with practice time, the relationship was in the expected direction, and it is likely that we were not powered to detect this relationship.

These data also indicate that improvements in sleep are associated with connectivity changes between the DMN and regions important for emotion, attention, and social cognition. Previous studies have shown that disordered sleep is related to altered brain function in the DMN as well as the SN [67-69]. Here, using principal components decomposition, we found that a small number of connections among DMN, SN, and dorsal attention network components, and with superior temporal, fusiform, and orbitofrontal areas, are closely associated with one another and with the explained variance of the delta-connectivity matrix. This area, explicitly characterized as i6-8, is functionally distinct from regions involved in simple eye movements and is considered part of a core complex involved in working memory, along with the inferior parietal sulcus [51]. The finding that changes in this network of regions are related to changes in sleep impairment further bolsters the existing evidence that the DMN and SNs are affected by or involved in sleep impairment.

PA well-being is a complex and multifactorial issue. Isolation, sleep deprivation and disturbance, and feeling overwhelmed by the amount of material they need to master are risk factors for depression among health care trainees [65,70]. Moreover, lack of time for self-care and stigma toward treatment-seeking are barriers to addressing mental health crises among trainees [2,71]. For these reasons, it is unlikely that a short-term, app-delivered mindfulness meditation program will be a stand-alone solution. Rather, addressing trainee mental health must be comprehensive and should include structural and organizational solutions alongside individualized resilience programming. Moreover, wellness programs for trainees must be sustainable and preventive in nature rather than reactive [64,72]. Medical training programs must make wellness feasible within the lives of trainees, and app-delivered programming may be a feasible and sustainable piece to foster a culture of resilience among PA students.

### Limitations

This study had a small sample, and the findings may not be representative of all PA students. We were likely underpowered to detect small effects, and the changes in sleep impairment did not reach significance at alpha levels adjusted for multiple comparisons. Moreover, it is unclear whether improvements in sleep impairment reported by students randomized to mindfulness are enduring. There is some evidence that there are sex differences in how disordered sleep affects brain function [73]. The students in our sample were primarily women, and thus, the results may not be generalizable to male trainees. Despite these limitations, the methods used here are a novel approach to understanding sleep impairment and a mindfulness intervention that may improve it, and these data indicate that app-delivered mindfulness may be effective for PA students.

## Appendix

Multimedia Appendix 1 Quality control and assurance measures, including motion correction and signal change assessments, validity of subject-to-stereotactic space registrations, effects of denoizing on connectivity, and functional connectivity as a function of distance from the seed voxel.

Multimedia Appendix 2 Supplementary figures and tables, including results of multivoxel pattern analysis, connectivity matrices by group and visit, and additional regression and classification results.

## Abbreviations

ANOVA: analysis of variance
DMN: default mode network
FDR: false discovery rate
fMRI: functional magnetic resonance imaging
GLM: general linear model
PA: physician assistant
PROMIS: Patient-Reported Outcomes Measurement Information System
ROI: region of interest
rsfMRI: resting-state fMRI
SI: sleep-related impairment
SN: salience network
TR: repetition time interval
