# Supplementary material for: Impact of App-Delivered Mindfulness Meditation on Functional Connectivity, Mental Health, and Sleep Disturbances Among Physician Assistant Students: Randomized, Wait-list Controlled Pilot Study
Source: JMIR Form Res. 2021 Oct 19;5(10):e24208. doi: 10.2196/24208 (PMC8564666; doi:10.2196/24208)
Supplement: Multimedia Appendix 1 [file formative_v5i10e24208_app1.pdf]

# Quality Control and Assurance

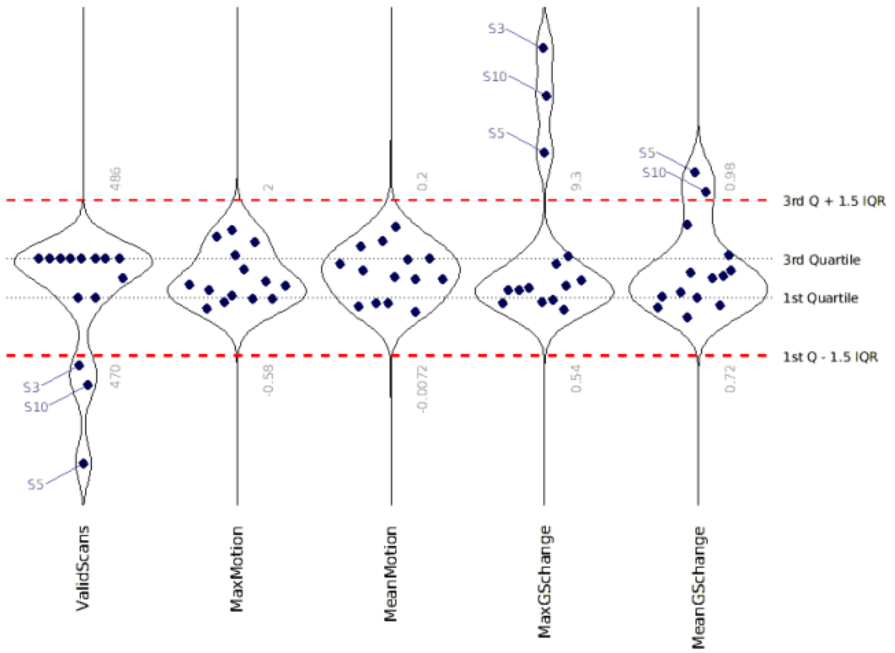


▲ Violin plots of the number of valid scans (*ValidScans*), mean and maximum motion (*MeanMotion*, *MaxMotion*), and mean and maximum global signal change (*MeanGSchange*, *MaxGSchange*) across all subjects. Outliers are annotated by subject ID; confidence intervals ($\pm 1.5 \times IQR$) are denoted by red dashed lines.

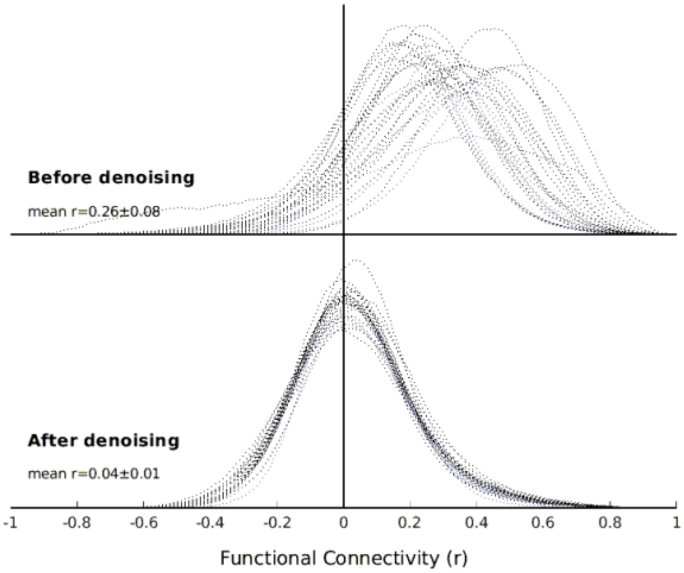


▲ Distribution of functional connectivity ($r$) across all subjects before and after denoising workflow.

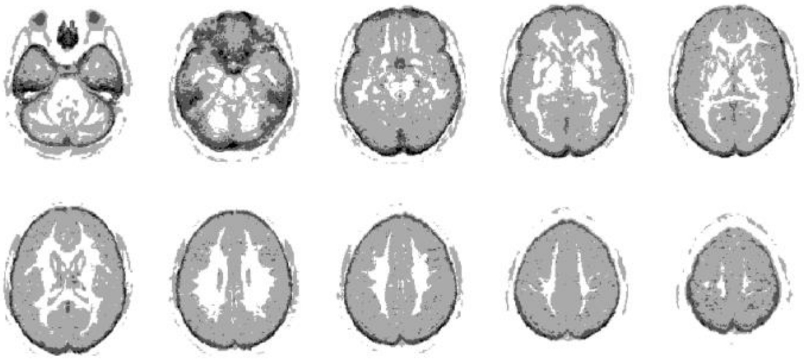

▲ Variability of registrations across all subjects following DARTEL registration versus outline of MNI-space grey matter template (*greyscale*).

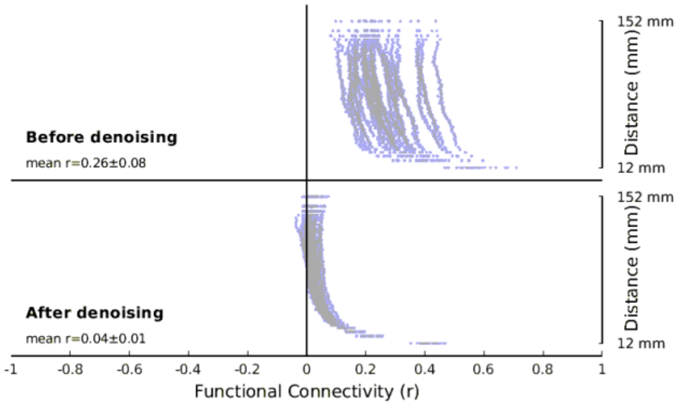


▲ Average functional connectivity ($r$) per voxel versus distance from the voxel, across all subjects, before and after denoising workflow.
