# Supplementary material for: Impact of App-Delivered Mindfulness Meditation on Functional Connectivity, Mental Health, and Sleep Disturbances Among Physician Assistant Students: Randomized, Wait-list Controlled Pilot Study
Source: JMIR Form Res. 2021 Oct 19;5(10):e24208. doi: 10.2196/24208 (PMC8564666; doi:10.2196/24208)
Supplement: Multimedia Appendix 2 [file formative_v5i10e24208_app2.pdf]

SUPPLEMENTARY MATERIAL

Clusters indicated by two-way ANOVA on PCs 1 and 2

Figure SF1. Two principal components were extracted for each voxel from subjects' within-visit, reduced-dimensionality dataset, then subjected to two-way ANOVA [*group* × *visit*] to select voxels which modulated with either condition (multivariate pattern analysis, MVPA). 87 voxel clusters, shown here, exhibited statistically-significant *group* × *visit* differences at $p_{FDR} \leq .05$. Of these, 16 clusters were excluded due to their localization in white matter. The 71 extant clusters were combined with atlas-based regions of interest (ROIs) corresponding to the default mode, sensorimotor, visual, salience, dorsal attention, frontoparietal, language, and cerebellar intrinsic connectivity networks for a total 102 ROIs. The corresponding ROI-ROI connectivity matrix was leveraged for further statistical analysis.

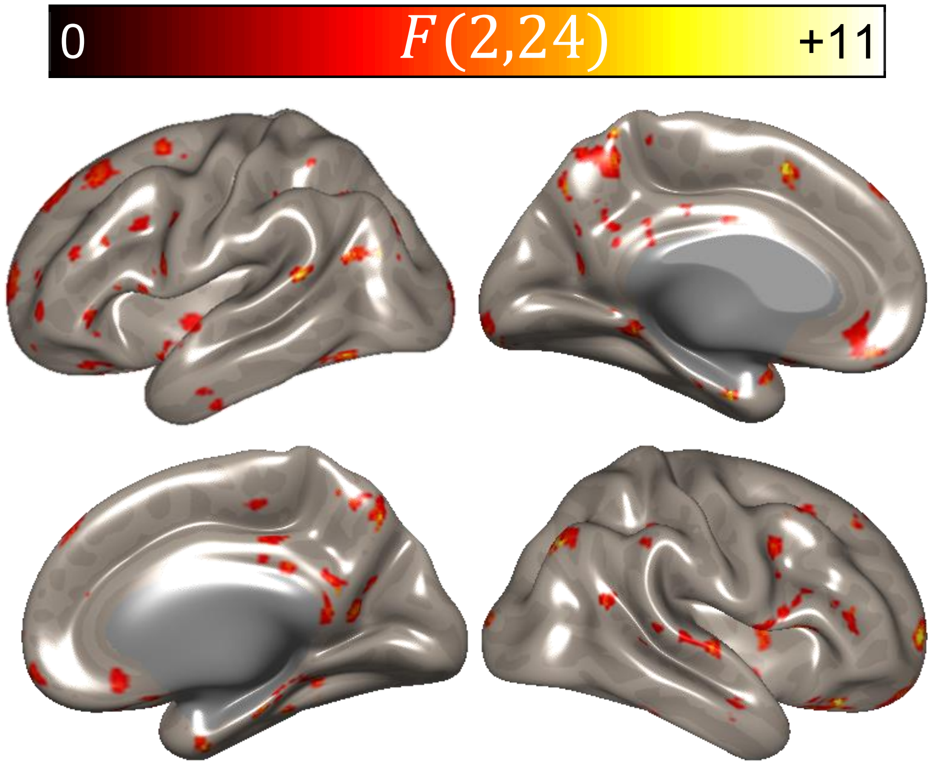

SUPPLEMENTARY MATERIAL

ROI-ROI connectivity matrices by group and visit

Figure SF2. Average ROI-ROI connectivity matrices by *group* (wait list or mindfulness) and *visit* (baseline or +8 weeks). A "delta matrix," **Δconn**, was also computed for each *group* as **Δconn** $= \mathrm{conn}_{8\mathrm{wks}} - \mathrm{conn}_{\mathrm{baseline}}$. Color bars below figure represent Pearson product-moment correlations between ROI pairs.

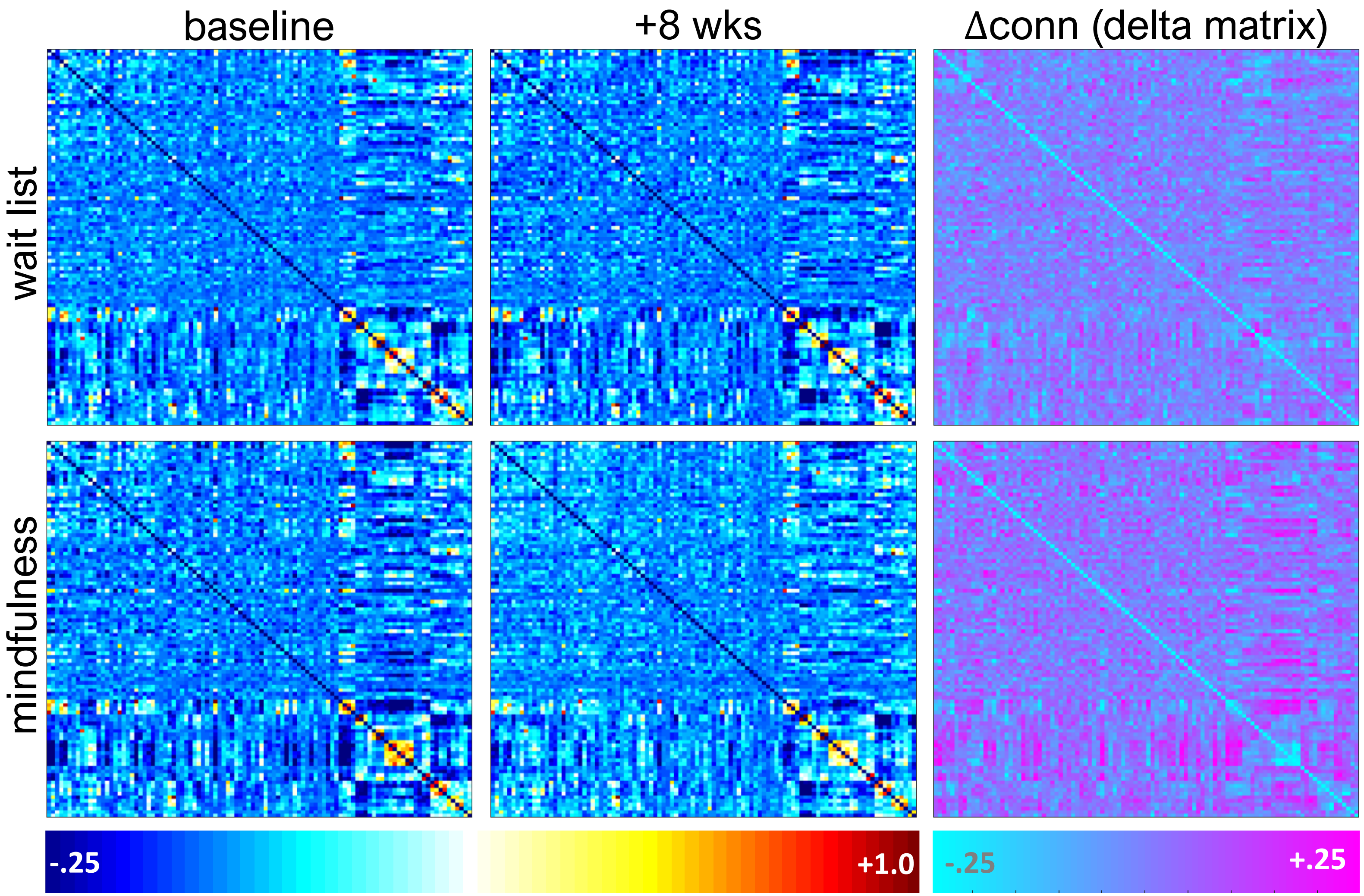

SUPPLEMENTARY MATERIAL

Table ST1. Results of linear regression on delta matrix with practice time as the predictor.

ID1	ID2	ROI1	ROI2	F	MSE	model p	post hoc	mean difference	p
op1_Rro	t5_Lba22c	R rolandic operculum/r. BA6	L sup. temp. visual area	22.3719	0.0092	0.0052	(intercept)		0.0052
anterior cerebellum	posterior cerebellum	anterior cerebellum	posterior cerebellum	9.5701	0.0068	0.027	(intercept)		0.0068

Table ST2. Results of one-way ANOVA on delta matrix with practice time **bin** as the predictor. Post hoc analysis by two-tailed t-test. p-values FDR-corrected by Benjamini-Hochberg procedure. Two-way ANOVA on group x practice time bin was not significant.

ID1	ID2	ROI1	ROI2	F	MSE	model p	post hoc	mean difference	p
cing1_Rpcc	f1_Lba9	**R post. cingulate ctx.**	**L sup. frontal g./BA9**	3.9467	0.0064	0.0405	practice Q2 < Q1	-0.2874	0.0354
pl2_Lba7m	orb1_Rba11	**L med. BA7/med. BA5**	**R lat. BA11/area Fo3**	3.9042	0.0316	0.0416	*n.s.*		
f3_Rba8v	pl3_Lba7m5ml	**R ventral BA8/IFJ**	**L med. BA7/med. lat. BA5**	5.6844	0.0213	0.0145	practice Q2 < control	0.5025	0.0132
							practice Q2 < Q4	0.5349	0.0324
							practice Q2 < Q1	-0.5702	0.0228
t1_Lffc	orb2_Lba10r14m	**L fusiform face complex**	**L r. BA10/med. BA14**	8.078	0.0124	0.0048	practice Q4 > Q3	-0.434	0.0061
							practice Q2 > Q3	-0.3428	0.0251
f9_Lba44v	orb4_L47l	**L ventral BA44/IFG tri.**	**L lat. BA47/IFG orb.**	6.129	0.0153	0.0116	practice Q1 > Q3	-0.5765	0.0264
							practice Q2 > Q3	-0.7354	0.0061
							practice Q3 < control	-0.5637	0.0137
op1_Rro	t5_Lba22c	**R rolandic operculum/r. BA6**	**L sup. temp. vis. area/BA22**	5.6448	0.0098	0.0149	practice Q4 < control	0.3135	0.0215
							practice Q4 < Q2	-0.3368	0.0474
							practice Q4 < Q1	-0.4284	0.0126
o3_Rv2v3d	t12_Rba38l	**R sup. occip. g./visual V2-V3**	**R dorsal area TG/BA 38**	4.4711	0.0079	0.029	practice Q3 > control	0.3558	0.029
f11_Rba6ba8	f12_Lba6ba8	**R sup. frontal g./sup. BA6-8**	**L mid. frontal g./inf. BA6-8**	4.3477	0.0115	0.0313	practice Q3 > control	0.3831	0.0516
							practice Q2 < Q3	0.4368	0.0528
f12_Lba6ba8	orb8_Rofc	**L mid. frontal g./inf. BA6-8**	**R OFC/area Fo2**	4.0289	0.0023	0.0384	practice Q3 < control	-0.1857	0.0356
							practice Q3 < Q1	-0.2011	0.0474

SUPPLEMENTARY MATERIAL

Figure SF3. Results of linear regressions with connectivity as the dependent variable and practice time (in minutes) as the predictor. Connections assessed included orbitofrontal MVPA ROIs *orb1* (Brodmann area 11) and *orb9* (Brodmann 14) and atlas-defined ROIs occipitopolar visual cortex, right lateral visual cortex, and left lateral sensorimotor cortex.

NOTE: MINDFULNESS group only

	vs	rho	p(FDR)
orb1_Rba11	orb9_Rba14m	0.991	0.0397
occipitopolar visual	R inferior parietal s. (DAN)	0.991	0.0397
R lateral visual	R inferior parietal s. (DAN)	0.991	0.0397
L lateral sensorimotor	orb9_Rba14m	-0.991	0.0397

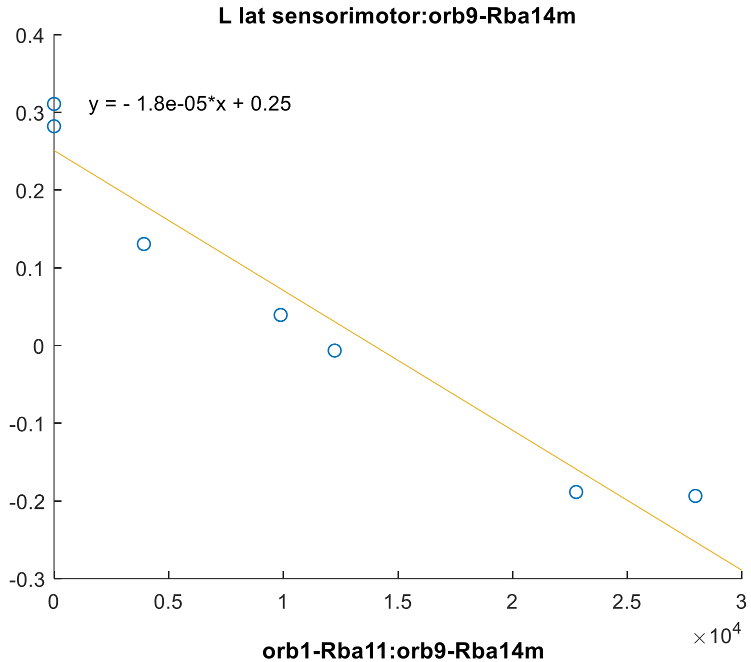

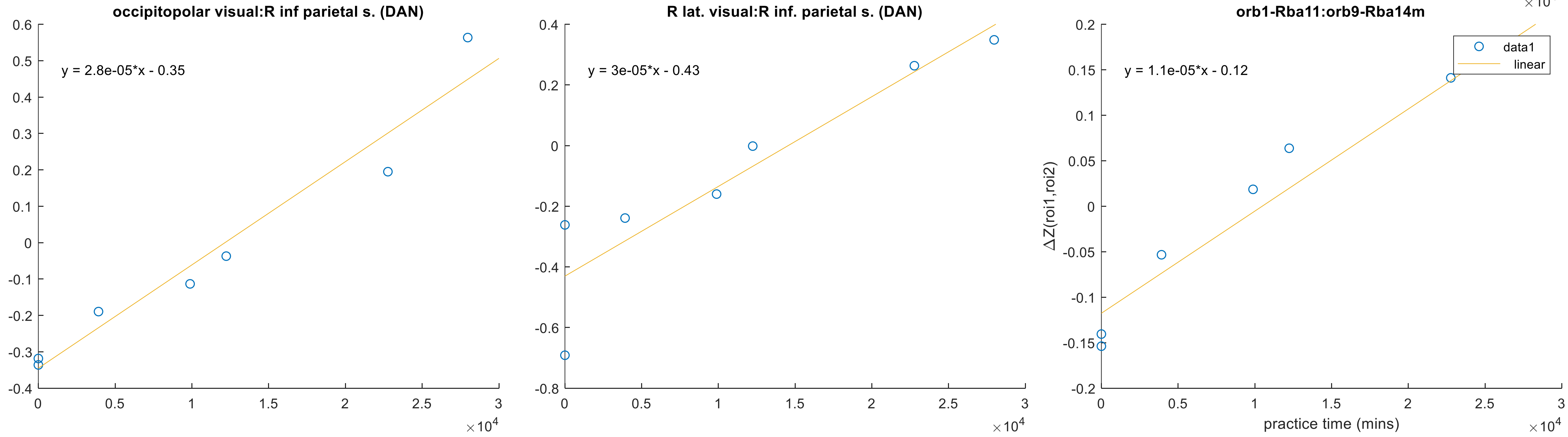

Predicting HAS_IMPAIRMENT=1 → HAS_IMPAIRMENT=0

Figure S4. Sleep impairment by subject and visit (visit 1, baseline; visit 2, +8 weeks) based on PROMIS scores at each visit. Three wait list subjects and three mindfulness subjects were classified as sleep-impaired at visit 1. All three mindfulness subjects (including one non-compliant subject) were classified as non-impaired at +8 weeks, whereas all three control subjects remained impaired.

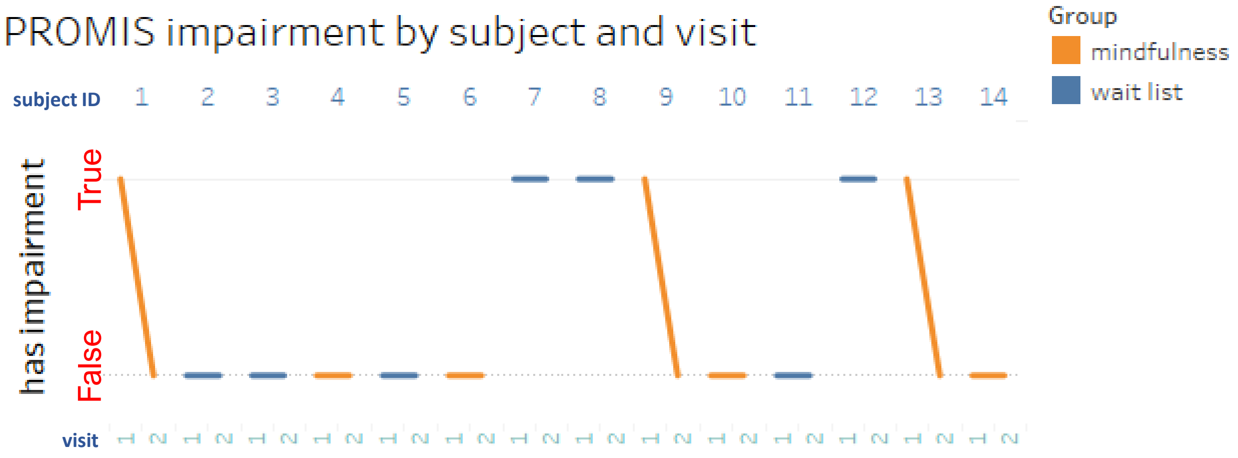

SUPPLEMENTARY MATERIAL

Table ST3. Classifiers used for prediction of change in IMPAIRMENT, with hyperparameters.

	scikit-learn class	hyperparameters tuned	train/test split	crossvalidation	tuning iterations
**support vector**	svm.SVC()	C; gamma; kernel (linear, polynomial, radial basis function, sigmoid); degree for polynomial (2-10); class weight = balanced or none; decision function = one versus one or one versus rest.	30:70	5-fold	5000
**gradient boosting**	ensemble.GradientBoostingClassifier()	learning rate; min samples to split (10-90%); max depth (1-100); number of estimators (10-1000)	30:70	5-fold	5000
**random forest**	ensemble.RandomForestClassifier()	min samples to split (10-90%); max depth (1-100); number of estimators (10-1500)	30:70	5-fold	5000
**multilayer perceptron**	neural_network.MLPClassifier()	hidden layer sizes (3-9 layers, 4-169 nodes per layer); activation function (logistic, tanh, ReLU); alpha (1e-5 to 5e-5); early stopping (true/false); solver (lbfgs, sgd, adam)	30:70	5-fold	5000
**gaussian naïve bayes**	naïve_bayes.GaussianNB()	smoothing parameter	30:70	5-fold	5000
**linear discriminant**	discriminant_analysis.LinearDiscriminantAnalysis()	solver (svd, lsqr, eigen); shrinkage (none or auto)	30:70	5-fold	5000

SUPPLEMENTARY MATERIAL

Table ST4. Results of change in IMPAIRMENT predictions using entire connectome (top: "no PCA decomp.") and using dimensionally-reduced connectome data (bottom: "with PCA decomp.").

		best parameters	accuracy	sensitivity	specificity	precision	FPR	FNR
no PCA decomp.	**support vector**	C=20.5316, balanced, OVR, degree=3, gamma=.0028, sigmoid kernel	77.14±7.82%	1.00	0.00	0.77	1.00	0.00
	**gradient boosting**	380 estimators, 0.1 min samples split, max depth=93, learning rate=.5	74.28±6.39%	0.97	0.00	0.77	1.00	0.03
	**random forest**	725 estimators, 0.70 min samples split, max depth=87	77.14±7.82%	1.00	0.00	0.77	1.00	0.00
	**multilayer perceptron**	solver=sgd, hidden layer sizes=(169,169,50), alpha=.0041, logistic activation, no early stopping	77.14±7.82%	1.00	0.00	0.77	1.00	0.00
	**gaussian naïve bayes**	smoothing=1.0239e-10	77.14±7.82%	1.00	0.00	0.77	1.00	0.00
	**linear discriminant**	lsqr solver, auto shrinkage	77.14±7.82%	1.00	0.00	0.77	1.00	0.00
with PCA decomp.	**support vector**	C=43.4765, balanced, OVR, degree=2, gamma=.0489, poly kernel	80.00±7.82%	0.20	0.93	0.33	0.07	0.80
	**gradient boosting**	300 estimators, 0.5 samples split, max depth=38, learning rate=.1	60.71±7.14%	0.00	0.87	0.00	0.13	1.00
	**random forest**	705 estimators, 0.55 samples split, max depth=22	82.85±6.39%	0.00	1.00	0.00	0.00	1.00
	**multilayer perceptron**	solver=lbgfs, hidden layer sizes=(169, 128, 64, 32, 16, 4), alpha=.0072, logistic activation, no early stopping	80.00±12.78%	0.00	0.83	0.00	0.17	1.00
	**gaussian naïve bayes**	smoothing=2.1142e-10	82.85±6.39%	0.00	1.00	0.00	0.00	1.00
	**linear discriminant**	lsqr solver, auto shrinkage	60.71±7.14%	0.25	0.83	0.11	0.17	1.00
